# A Serious Game on the First-Aid Procedure in Choking Scenarios: Design and Evaluation Study

**DOI:** 10.2196/16655

**Published:** 2020-08-19

**Authors:** Imma Boada, Antonio Rodriguez Benitez, Santiago Thió-Henestrosa, Josep Soler

**Affiliations:** 1 Graphics and Imaging Laboratory Escola Politècnica Superior, Edifici Politècnica IV 17003 Girona Spain

**Keywords:** choking, prevention, first-aid procedure, first-aid education

## Abstract

**Background:**

Choking is one of the causes of unintentional injury death. Gaining the knowledge of the first-aid procedure that has to be applied in case of choking can increase the chances of survival of persons with choking. Serious games can be a good channel for educating people about choking scenarios and the actions to be taken to save the persons with choking.

**Objective:**

The objective of this study is to present and evaluate the effectiveness of a serious game designed to prevent choking and to promote the first-aid procedure that needs to be applied in case of choking.

**Methods:**

In this study, we present a serious game as a set of minigames that reproduces the main steps of the protocol for the first-aid performed in choking. In the proposed game, the player acquires the role of a helper who has to save the person in a choking emergency by applying the main steps of the protocol. Time and score restrictions are imposed to pass each minigame. To test this game, we performed a pilot study with 48 high school students. Different tests were performed to assess the students’ preferences and their knowledge on choking before and after playing the proposed game. The obtained results were analyzed using Mann-Whitney *U* test when a grade variable was involved and by using Fisher exact test when 2 categorical variables were involved.

**Results:**

The findings of our study showed that the players enjoyed the game. No statistical differences were detected when considering the gender of the player, their preferences for video games, or their previous experience in choking emergencies. By comparing the knowledge of these students before and after playing the game, we found that all the indicators of the knowledge about how to act in case of a choking emergency were improved through this serious game.

**Conclusions:**

The findings of our study show that the proposed game is a good strategy for promoting and teaching first-aid procedures in choking emergencies to nonexperts in this field.

## Introduction

A foreign object lodged into the throat or the windpipe may cause choking. Unless the air passage is cleared, the person with choking can lose consciousness within 3-5 minutes. In worse cases, the lack of oxygen to the brain could cause brain damage or death. In these situations, it is recommended to administer, as quickly as possible, first-aid that consists of abdominal thrusts combined with back blows [1]. Unfortunately, not everyone is aware of this procedure, and the strategies to promote the awareness of this first-aid are necessary [2].

In order to educate about the risks, prevention, and treatment of choking, different approaches have been proposed. Organizations such as the Resuscitation Council [3], the Red Cross [1], or the American Heart Association [4] have defined guidelines that describe how choking interventions should be performed both safely and effectively. These organizations also offer courses, videos, and other materials to teach the procedure of the choking rescue. In addition, many countries have initiated campaigns to educate their citizens about the prevention, risks, and treatment of choking. Special attention has been given to children since they are the most susceptible to choking [5]. The effectiveness of these initiatives has also been studied and, in most of the cases, the general conclusion is that prevention strategies are effective and contribute to decreasing the choking incidence [6-8]. Moreover, several initiatives have focused on the development of first-aid training devices. The Zoll Medical Corporation has proposed a handheld device that uses several accelerometers to monitor abdominal thrusts [9]. Recently, Watson and Zhou [10] presented BreathEZ, a smartwatch app that provides both first-aid instructions for choking and real-time tactile and visual feedback on the quality of the abdominal thrust compressions.

In this paper, we propose to tackle the problem of choking by using serious game strategies. Serious games are digital games used for purposes other than mere entertainment and are applied in different areas such as military, government, education, and health care [11]. They can recreate scenarios to experiment with situations that otherwise would be impossible in the real world owing to the required safety, cost, and time [12]. In addition, serious games enhance the development of skills such as analytical and spatial, strategic, or psychomotor [13]. Although there are a large number of serious game apps related to health care, as far as we know, there are no serious games focused only on first-aid education for choking.

Serious games have become a powerful tool to develop and acquire new knowledge and skills. Different games have been developed for health care purposes and tested in a wide range of diseases, treatments, and other related topics [14-16]. These games focus not only on expert users but also on nonexperts.

Baranowski et al [17] presented a classification of games for health in 5 categories. One category, which was centered on health care professionals, consists of games designed to provide simulation environments and virtual patients to practice and acquire relevant skills [18]. An example in this category is MyCraft, which provides virtual consultation training on tuberculosis [19] and games to practice surgeries, blood management, image-guided procedures, assessment, prevention, and treatment [20-24]. The other 4 categories, which are centered on nonexperts such as patients or general users, consist of games designed to increase knowledge, change behavior, or involve health behavior in gameplay. An example of a game that increases knowledge and changes behavior is Yummy Tricks, which is a game intended to teach healthy eating habits [25-27] or the game by Ito et al, which was developed to evaluate the dissemination of public awareness on preschool children’s oral health [28,29]. Regarding games that involve behavior, exergames are the most representative of this category. These games incorporate physical activity in the gameplay and can be used for different purposes such as physical activity encouragement [30-33] or rehabilitation to recover from brain injuries, cognitive impairments [34], or motor deficiencies [35,36]. Finally, with respect to the games that influence health precursors, some examples are games that reduce stress and anxiety [37], deal with depression [38], or prepare for cancer treatments [39]. The game proposed in this paper can be classified as a game designed for nonexpert users to increase their knowledge on a health topic, particularly on first-aid survival techniques.

In the context of the first-aid techniques, different serious games have been proposed. The Virtual Heroes company [40] presented serious games for health care professionals, such as 3DiTeams [41], a first person, multiplayer training app, wherein the player is placed in a high-fidelity virtual hospital; Combat Medic [42], a web-based 3D collaborative virtual world to deal with hemorrhage, airway management, and tension pneumothorax; and HumanSim:Blast [43], wherein after a train station explosion, the player must identify and label zones on an area map, tag potential hazards, assess patient vitals, perform life-saving procedures, and triage patients. In a similar way, the BreakAway Company proposed Code Orange [44], a serious game wherein the players work in concert with the first-aid staff of a hospital to save people injured in a weapon of mass destruction event. Other games for emergency staff training are Nuclear Event Triage Challenge [45], Peninsula City [46], Burn Center [47], and CliniSpace [48]. In the field of cardiopulmonary resuscitation (CPR), Jerin et al proposed the automated external defibrillator challenge, which is a web-based serious game for teaching and training automated external defibrillation and first-aid maneuvers to lay people and emergency medical service professionals [49]. Other proposals are JUST [50], an immersive virtual reality situation training system for nonprofessional health emergency operators; MicroSim Prehospital [51] designed for prehospital training on emergency medical services; and Staying alive [52], an online 3D simulator that provides a learning experience of saving a virtual patient from cardiac arrest in 4 minutes; LISSA [53,54], which presents an emergency situation wherein CPR actions have to be applied to save the person with choking; 30:2 [55], a game designed to educate on CPR protocol to nonexperts; Relive [56], a first person 3D adventure where the player faces different rescue situations; Viva!Game [57], a web-based serious game designed to create awareness on cardiac arrest and CPR; and HeartRun [58], a mobile simulation game to train resuscitation and targeted at giving school children an understanding of this protocol. Recently, Benkhedda and Bendella [59] presented FASim, a 3D serious game that combines health care simulations with serious games and the functionalities of the multiagent systems in a single framework in order to learn the first-aid procedure for and the signs of a cardiac arrest. In the context of choking, Carvalho et al [60] proposed an Android app video game wherein different first-aid actions are presented to familiarize users with these scenarios, choking being one of them.

In most of the cases, the games focused on first-aid procedures have been designed for patients with health issues and the game being tailored to address their health issues adequately [61]. In general, little attention has been paid to nonexperts, although first-aid protocols would be a basic knowledge for everyone. To overcome this limitation, our aim was to exploit the advantages of serious games and use them to promote basic knowledge on choking recovery procedures. Regarding choking procedures, no games related to this topic have been proposed and this is the novel aspect of our approach. The advantages of game-based technologies over traditional education methods have been studied by many authors [62,63]. Game-based technologies are more effective because they use action instead of explanation, they are able to create personal motivation and satisfaction and accommodate multiple learning styles and skills, and they are able to provide an interactive and a decision-making context [64,65]. These facts combined with the extended use of portable gaming platforms makes computer-based games a perfect channel to promote learning contents [66] such as the ones required to educate about the choking procedure.

## Methods

### Main Design Decisions

**Figure 1:**
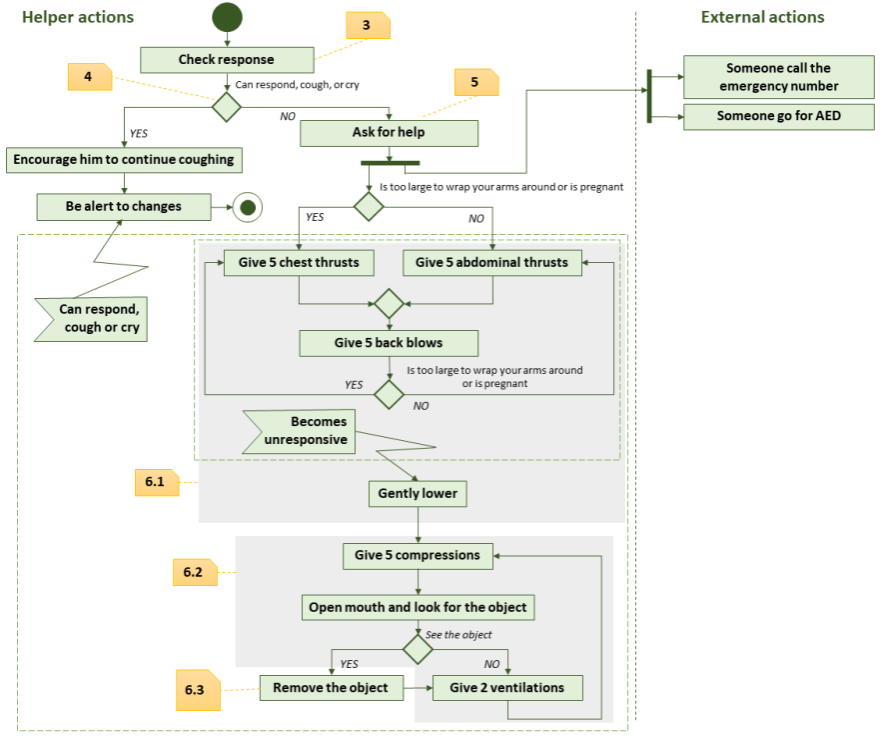
Main steps of the first-aid procedure that has to be applied in a choking emergency. The numbers in the boxes indicate the relationship of the minigames 3, 4, 5, and 6 with the procedure steps. AED: automated external defibrillator.

In this section, we present the main considerations that have been taken into account to design the game. To create this game, the educational game development approach proposed by Torrente et al [67] was applied. This approach covers all the tasks of the game design from implementation to evaluation. It is built on 4 basic principles: (1) the procedure-centric approach that gives importance to capturing the procedural knowledge of the domain, (2) the collaboration between experts, (3) the agile development with agile tools, and (4) the low-cost game model. In our case, these principles were applied as follows. The game procedural knowledge of the domain is given by the choking protocol illustrated in Figure 1. This knowledge was supervised by the group of physicians that collaborated with our research group. The agile development was done via an iterative design and a development process that included analysis, game design, implementation, and quality assurance. Finally, instead of the 3D realistic models, our game is based on 2D animations, thereby leading to a low-cost game model.

The pedagogical approach used to design the game was based on the experiential learning theory, wherein educators aim at engaging learners in direct experience to increase their knowledge, skills, and values [68]. Experience occurs as a result of interaction between human beings and the environment in the form of thinking, seeing, feeling, handling, and doing [68]. In our case, this experience is going to take place in an artificial environment wherein a choking person has to be identified by the choking symptoms and also recovered by trying to reproduce the protocol. There are 5 instructional strategies rooted in the concept of learning through experiences. These are learning by doing, experiential, guided experiential, case-method teaching, and a combination of experiential and inquiry-based learning [64]. In our case, the proposed approach can be seen as a learning by doing strategy. The idea is to reproduce the steps of the choking protocol several times. Since just doing actions does not involve acquiring the knowledge, the game will also include instructions and feedback messages to make the actions meaningful in order to consolidate the player knowledge [69].

The last issue to be considered was how to deal with the protocol steps. From our experience in the previous games that focused on first-aid protocols [55,70], we decided to decompose the game into a set of minigames, thereby making knowledge acquisition easy for the players [71]. Therefore, the game in this study is composed of 6 minigames, the player being the first one to put in context, then presenting the elements that can cause choking, and the rest of the game is focused on the steps of the choking recovery procedure (Figure 1). The main steps of the first-aid procedure have to be applied in a choking emergency. The numbers in the boxes indicate the relationship of the minigames 3, 4, 5, and 6 with the procedure steps.

### Choking Prevention Game

The 6 minigames that compose the game can be seen as submissions to reach the goal, which is to save a person from choking by applying the steps of the choking protocol. The submissions are designed to identify the choking symptoms or to apply a specific step of the protocol. In all the minigames, the player acts as a rescuer who interacts with the main character that represents the person with choking. This character appears in all the minigames in a similar scenario, with the same screen design, with time and score information on the top of the screen, as well as help messages used to highlight the relevant information of the step. To guide the player between the minigames, at the beginning of each minigame, the instructions of the step protocol are presented. There is also a help icon to access an animation that describes it. In addition, at the end of the minigame, another message communicates if the step has been achieved or not.

In all the minigames, correct actions add points and incorrect ones subtract points. The minigame is finished when the time is finished or when the maximum score is reached. Two playing modes are supported. The player can play each minigame independently to reach the maximum level or sequentially, thereby completing all the sequences of minigames with the same level of difficulty, to see the whole protocol each time. All user interactions are limited to touch and drag-and-drop actions. In this way, the players can achieve great mastery within a short period. A detailed description of each minigame is given below. For each one, first, the learning objective is presented, and then the design is proposed to achieve it. A complete demo of the game is provided on https://youtu.be/cABGCo7R2HI.

#### Minigame 1: Choking Prevention

**Figure 2:**
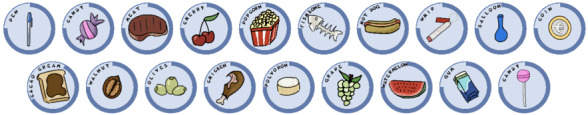
Some of the items of the choking prevention minigame, wherein player actions vary according to the type of item.

**Figure 3:**
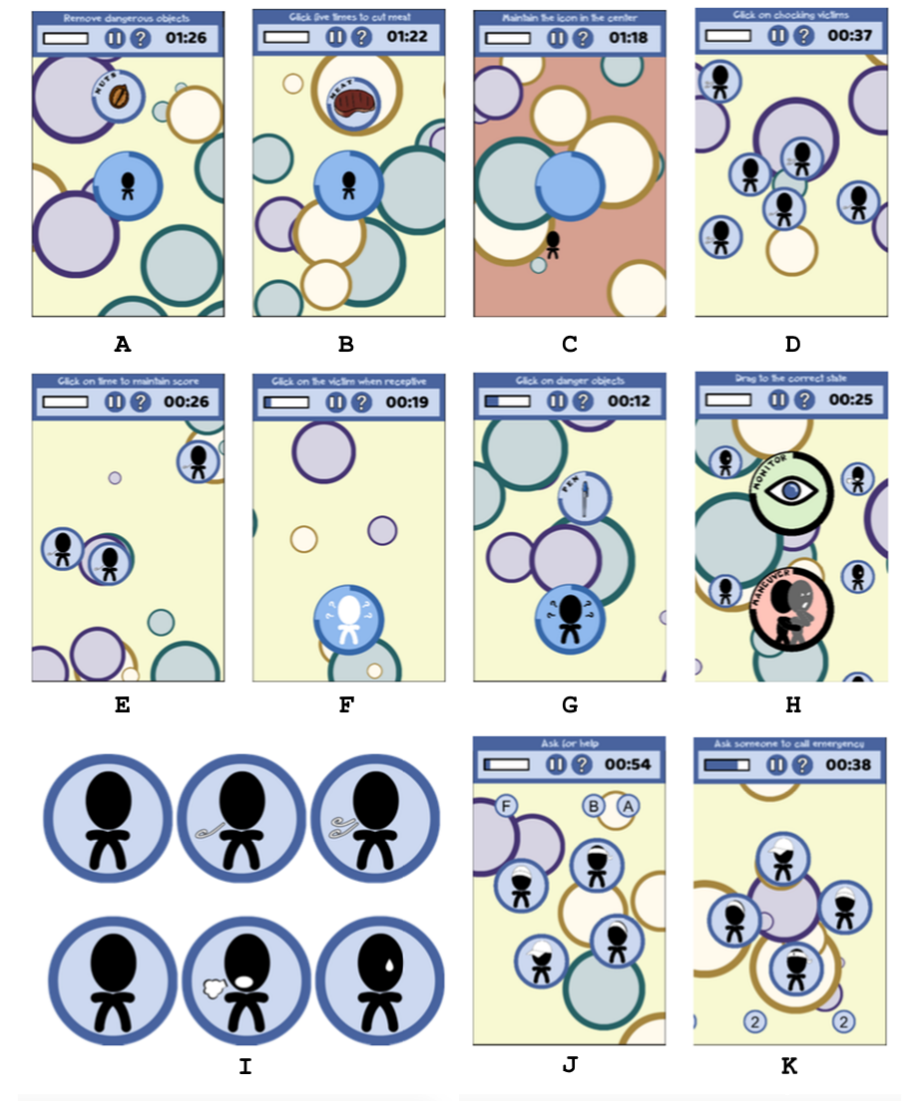
Choking prevention minigame. A. Avoid small and dangerous objects. B. Keep food pieces small. C. Do not move while eating. D and E. Identify choking persons minigame. F and G. Ask for response minigame. H. Identify choking symptoms minigame. I and J. Ask for help minigame. K. The icons representing breath status (no breath-breath with difficulties-normal breath) and choking symptoms (no breath-cough-cry).

In the first minigame, we focus on children who are more likely to choke than adults. The aim of this minigame is to promote 3 tips to prevent choking by using the following 3 methods: (1) avoid small and dangerous objects, (2) keep food pieces small, and (3) not move while eating. To reproduce these situations, an icon representing a child appears in the middle of the screen and different elements are placed around it. The player has to interact with these elements and carry out different actions according to the elements (Figure 2). In particular, if it appears to be a dangerous element, the player has to drag it out of the screen. If it appears to be a piece of meat, the player has to click on it 5 times to simulate cutting of small pieces. If the child icon moves from the center, the player has to drag it to the center again. This last action represents not to move while eating. In Figure 3A-3C, the different scenarios corresponding to these situations are illustrated.

#### Minigame 2: Identify Persons with Choking

The aim of this minigame is to identify persons with choking and remove them from the screen. On the screen appears person icons with 2, 1, or no air pathways to represent breath status as normal, with difficulty, and airway totally blocked, that is, choking person, respectively. When the icon is on the screen, its breath status can become worse (Figure 3D and Figure 3E). The player has to click on the choking persons and if these are not selected, they will disappear and the player score will decrease.

#### Minigame 3: Check Response

A choking person will not be able to talk but will probably communicate through signs and actions such as grabbing his or her throat. The rescuer has to know when the choking person is able to communicate or not. To reproduce this situation, in this minigame, a choking person represented as a person icon appears on the screen. The icon changes its color from black to white to indicate that the choking person is receptive to be asked or not, respectively. In addition, danger elements going to the choking person appear on the screen. The player has to drag these objects out from the screen and click on the choking person when the icon is receptive be asked. If a dangerous object arrives to the choking person, the icon cannot be asked and the player score will decrease. The score also decreases when no receptive choking person is asked. In Figure 3F and Figure 3G, screenshots of this minigame are presented.

#### Minigame 4. Identify Choking Symptoms

A choking person typically has a panicked, confused, or surprised facial expression and usually place hands on the throat. If the airway is not totally blocked, choking persons will be able to cough or make squeaking noises while trying to breathe. If the airway is totally blocked, the choking persons will not be able to speak, cry, or cough, and their skin color will range from red to pale owing to the lack of oxygen. In this minigame, person icons appear on the screen and some of them represent choking symptoms. The player has to identify icons and separate choking persons from the others. Points are lost in case of incorrect or nonclassification of choking persons. A screenshot of this minigame is presented in Figure 3H, and the icons representing the different symptoms are shown in Figure 3K.

#### Minigame 5. Ask for Help

After the identification of the person with choking and confirming the person with choking, the player has to ask for help and call an emergency number.

In this minigame, person icons as well as letters on the top and numbers on the bottom will appear on the screen. The player has to select one of the icons and write *help* by combining selected letters from the top. The icons and letters are continuously moving. The same procedure has to be done to call emergency numbers by selecting one of the icons, not necessarily the same, and attaching the correct emergency numbers. The player ends the game when *help* and the correct emergency number are written. The game screens are shown in Figure 3I and Figure 3J.

#### Minigame 6. Choking Maneuver

This minigame represents the most important part of the choking rescue protocol. To reproduce it, we have divided this minigame into 3 parts that recreate back blows and abdominal thrust, CPR, and object removal. To pass this minigame, the player has to pass the 3 parts. The description of each part is presented below.

##### Perform Back Blows and Abdominal Thrusts

**Figure 4:**
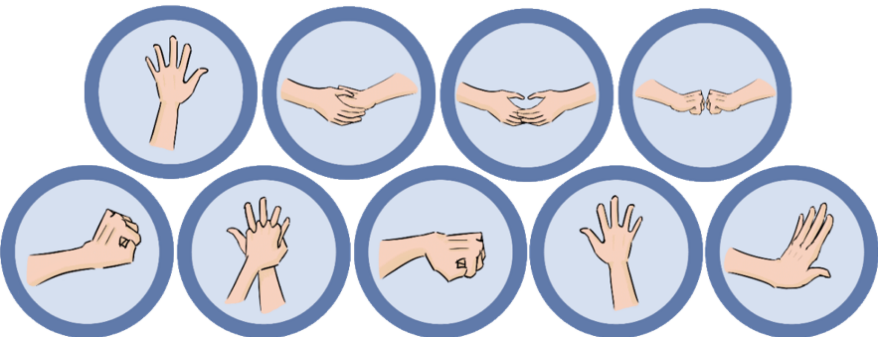
Icons representing different hand positions.

**Figure 5:**
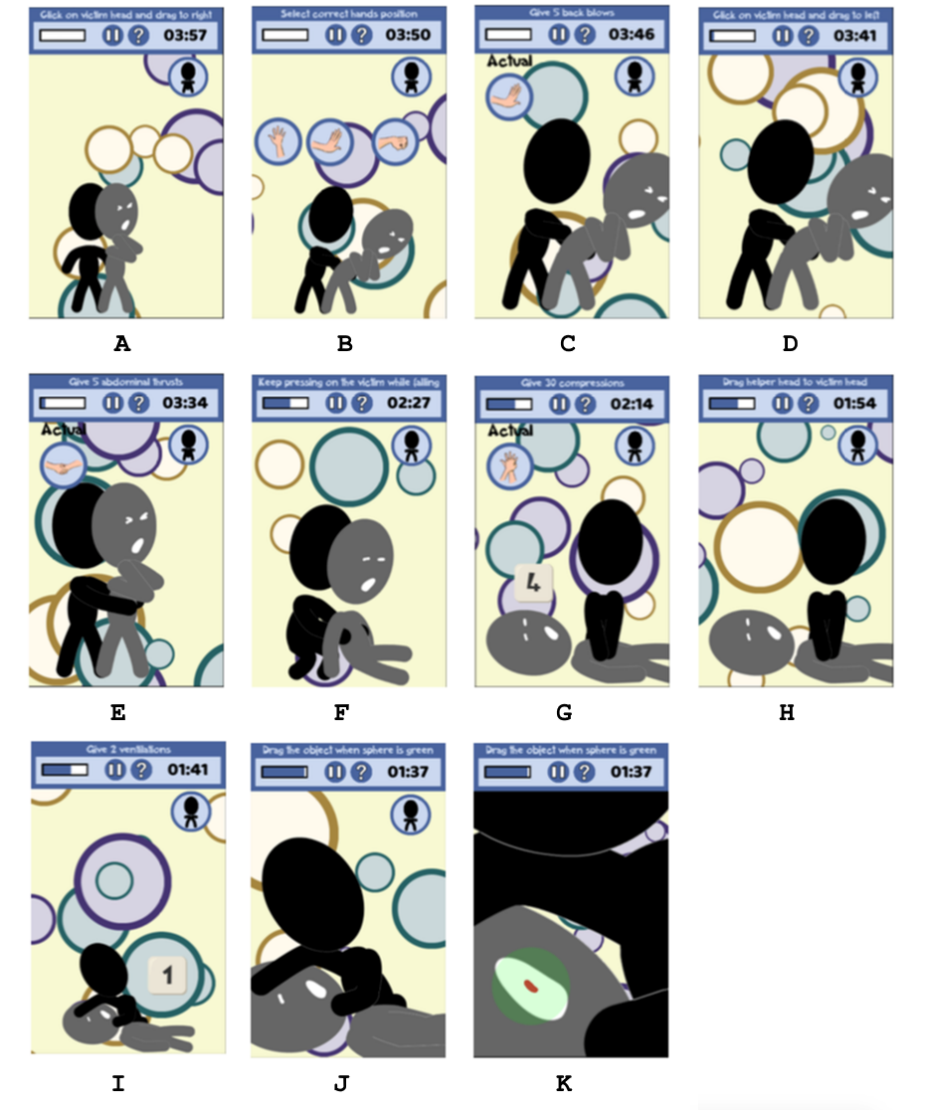
Choking maneuver screens corresponding to the different parts of the minigame. A-E. Back blows and abdominal thrusts. B, F, G, H, J. Cardiopulmonary resuscitation. I. Remove the object. K. Rescue breaths.

For adults and children with choking, to force the object out of the airway, the helper has to give a combination of 5 back blows between the shoulder blades followed by 5 abdominal inward and upward thrusts just above the navel. To reproduce this situation, 2 silhouettes representing the choking person and the helper and icons representing different hand positions will appear on the screen (Figure 4). First, the player has to put the choking person in the correct position by clicking on the head and dragging to the right side (Figure 5A) and then select the correct hand position represented in one of the different icons that will appear (Figure 5B). Then, the player has to give 5 clicks on the correct position of the choking person (Figure 5C) to simulate the 5 back blows. The player has to place the choking person in the initial position by clicking on the head and dragging to the left side (Figure 5D). The player has to perform 5 abdominal thrusts by selecting the correct position of the hands (Figure 5B) and then by clicking 5 times in the correct position (Figure 5E). This process is repeated until the choking person ejects the object or passes out. If the person ejects the object, a new choking person will appear. If the choking person passes out, the second part of the minigame, that is, CPR, will start.

##### Perform CPR

When the choking person is unresponsive because back blows and abdominal thrusts have been unsuccessful, the player has to perform the CPR, which combines 30 chest compressions with 2 rescue breaths. First of all, the player has to gently lower the choking person by touching the body (Figure 5F). At this point of the minigame, the choking person and the helper stay in the position of the CPR protocol. To start, the player has to select the icon representing the correct hand position to perform chest compressions. Then, the player has to click 30 times on the chest of the choking person following the correct rhythm (Figure 5G). After each set of chest compressions and before rescue breaths, the player has to click on the mouth of the choking person and look for the object. If the player sees the object, the third part of the minigame starts (Figure 5H). If the object is not visible, the player has to perform 2 rescue breaths by touching the mouth of the choking person following the correct rhythm (Figure 5K). Again, a new sequence of compressions has to be performed and the process is repeated until help arrives.

##### Remove the Object

If the helper sees the object in the mouth of the of the choking person, it has to be removed. To reproduce this situation, the object appears in the mouth of the choking person with a sphere behind it. The sphere changes its color and the player can only move the object when it is green (Figure 5I and Figure 5J). If the player moves the object when the sphere has another color, the object will fall. The object has to be removed in the given time.

### Designed Study and Statistical Analysis

To test the game, a sample population consisting of 48 high school students from a summer camp of our university was considered for this study. Our laboratory, the Graphics and Imaging Laboratory, participated in this camp for a 2-h session that was carried out in a computer laboratory. The aim of the session was to introduce high school students to video games and serious games. After the first introduction of our research, we asked the students to answer the questionnaire presented in Textbox 1. Then the students had 30 minutes to play the proposed game as an example of a serious game. Each student had a computer to play, and no introduction to the game was given; the students were just asked to play the game. During the session, we observed them so that we could detect the difficulties that the students encountered in the game. After the session, we asked them to answer the new questionnaire presented in Textbox 2.

To detect significant statistical differences, we used the Mann-Whitney *U* test when a grade variable was involved and we used the Fisher exact test when 2 categorical variables were involved.

**Test 1 questionnaire before playing the game.**

**Questionnaire to be completed before the game**

1. Select gender: Male/female

2. Age: Insert the number

3. Do you like video games? Yes/No

4. Do you know what is choking? Yes/No

5. Grade your knowledge of the choking first-aid protocol from 1 to 5 (1 being the least and 5 being the highest):

6. Have you ever been in a choking emergency? Yes/No

7. In case of choking, to remove the throat object you have to (select only 1 correct option):

- Perform abdominal thrusts until object expulsion
- Perform back blows until object expulsion
- Repeat 5 back blows and 5 abdominal thrusts until object expulsion
- Repeat 10 back blows and 10 abdominal thrusts until object expulsion.

8. In which of the following cases of choking, would the cardiopulmonary resuscitation (CPR) protocol be applied? (select only 1 correct option):

- In all the cases, as it is a part of the choking first-aid protocol
- In case of unconscious choking persons
- Never, as the object causing choking does not need to be removed by CPR.

9. Write the emergency number.

**Test 2 questionnaire after playing the game.**

**Questionnaire to be completed after the game**

1. Do you know what is choking? Yes/No

2. Grade your knowledge of the choking first-aid protocol from 1 to 5 (1 being the least and 5 being the highest):

3. In case of choking, to remove the throat object you have to (select only 1 correct option):

- Perform abdominal thrusts until object expulsion
- Perform back blows until object expulsion
- Repeat 5 back blows and 5 abdominal thrusts until object expulsion
- Repeat 10 back blows and 10 abdominal thrusts until object expulsion.

4. In which of the following cases of choking, would the cardiopulmonary resuscitation (CPR) protocol be applied? (select only 1 correct option):

- In all the cases, as it is a part of the choking first-aid protocol
- In case of unconscious choking persons
- Never, as the object causing choking does not need to be removed by CPR.

5. Write the emergency number.

6. Grade your experience of the game from 1 (completely disagree) to 5 (completely agree) for each of the following categories:

- The game is entertaining.
- I like the game.
- The complexity of the game is correct.
- The help indications provided by the game are enough.
- The game describes how to proceed in a choking emergency.
- After playing the game, I know how to proceed in a choking emergency.

## Results

### Categories of the Findings

The obtained results are presented in 3 parts. The first part describes the sample population, the second one describes the results obtained from the game performance evaluation, and the third one compares the results obtained before and after playing the game.

### Test Population

Our test population of 48 high school students consisted of 16 males (33%) and 32 females (67%). Their mean age was 15.42 (SD 0.74) years. Of these 48 students, 29 liked video games (60%) while 19 did not like video games (40%), and12 encountered a choking emergency (25%) while 36 never encountered this emergency situation (75%).

### Evaluation of the Game Performance

Firstly, we present the results obtained from the answers related to the game such as the complexity and the degree of entertainment (Test 2 question 6). Then, we describe the results considering the gender of the player, video game preferences, and choking emergency experience.

In Table 1, the descriptive statistics of the answers to the questions related to the game are shown. Although, the answers are not extremely positive, we can observe that they tend to be more positive than negative. Note that all the medians are above 3; the median of *the help indications provided in the game are enough* (3.8) was the highest followed by the medians of *the game describes how to proceed in a choking emergency* (3.58), *the game is entertaining* (3.5), and *the complexity of the game is correct* (3.46). These results show that the primary aim of the game, that is teaching the procedure that has to be applied in case of choking, was achieved. In addition, we saw that the players enjoyed the game; it was considered to be not boring and not difficult. Our results also show that the game effectively described how to proceed in a choking emergency.

In Table 2, the descriptive statistics of the answers to the questions related to the game based on the player gender are shown. No statistically significant differences were found except with regard to the complexity of the game. All the items showed a small effect size, except the complexity of the game, which showed a moderate effect size. Regarding the complexity of the game, more males than females agreed that that complexity was appropriate. The results based on whether the students liked video games or not are presented in Table 3, and the results based on whether the students had a previous experience of choking emergency are presented in Table 4. No statistically significant differences were found in any of the cases, and the effect size was small. Therefore, we can consider that this serious game on first-aid for choking fits the different player profiles.

**Table 1:** Descriptive statistics of the answers to the questions related to the game.

Answers to the questions (grades 1-5)	Median (Q1, Q3)
The game is entertaining	3.5 (3, 4)
I like the game	3.02 (2, 4)
The complexity of the game is correct	3.46 (3, 4)
The help indications provided by the game are enough	3.8 (2, 4)
The game describes how to proceed in a choking emergency	3.58 (2.75, 5)
After playing the game, I know how to proceed in a choking emergency	3.13 (2, 4)

**Table 2:** Descriptive statistics of the answers to the questions related to the game (grades 1-5) by each gender.

Answers, Gender		Median (Q1, Q3)		*P* value		Effect size	
**The game is entertaining**					.21		0.182
	Females^a^		4 (3, 4)				
	Males^b^		3 (2, 4)				
**I like the game**					>.99		0.0016
	Females		3 (2, 4)				
	Males		3 (2, 4)				
**The complexity of the game is correct**					.03		0.324
	Females		3 (3, 4)				
	Males		4 (3.75, 4.25)				
**The help indications provided by the game are enough**					.85		0.293
	Females		3 (2, 4)				
	Males		3 (2, 4)				
**The game describes how to proceed in a choking emergency**					.69		0.059
	Females		4 (2.75, 4.25)				
	Males		4 (3, 5)				
**After playing the game, I know how to proceed in a choking emergency**					.69		0.060
	Females		3 (2, 4)				
	Males		3 (3, 4)				

^a^Number of females in the study=32.

^b^Number of males in the study=16.

**Table 3:** Descriptive statistics of the answers to the questions related to the game (grades 1-5) depending on whether the students like video games or not.

Answers, Video game preference category		Median (Q1, Q3)		*P* value		Effect size	
**The game is entertaining**					.89		0.021
	Not like^a^		3 (3, 4)				
	Like^b^		4 (3, 4)				
**I like the game**					.17		0.198
	Not like		3 (2, 3)				
	Like		3 (2, 4)				
**The complexity of the game is correct**					.19		0.192
	Not like		3 (3, 4)				
	Like		4 (3, 4)				
**The help indications provided by the game are enough**					.64		0.069
	Not like		3 (2, 4)				
	Like		3 (2, 4)				
**The game describes how to proceed in a choking emergency**					.64		0.069
	Not like		4 (3, 4.5)				
	Like		3 (3, 5)				
**After playing the game, I know how to proceed in a choking emergency**					.36		0.133
	Not like		3 (2, 4)				
	Like		3 (3, 4)				

^a^Number of students who did not like playing video games=19.

^b^Number of students who liked playing video games=29.

**Table 4:** Descriptive statistics of the answers to the questions related to the game (grades 1-5) depending on whether the students had previously experienced a choking emergency.

Answers, Experience of a choking emergency		Median (Q1, Q3)		*P* value		Effect size	
**The game is entertaining**					.68		0.061
	No^a^		4 (3, 4)				
	Yes^b^		3.5 (2.75, 4)				
**I like the game**					.87		0.025
	No		3 (2, 3)				
	Yes		3 (2.75, 4)				
**The complexity of the game is correct**					.57		0.084
	No		4 (3, 4)				
	Yes		3 (3, 4)				
**The help indications provided by the game are enough**					.81		0.036
	No		3 (2, 4)				
	Yes		3 (2, 4)				
**The game describes how to proceed in a choking emergency**					.49		0.100
	No		4 (3, 5)				
	Yes		3.5 (2.75, 4.25)				
**After playing the game, I know how to proceed in a choking emergency**					.72		0.053
	No		3 (2, 4)				
	Yes		3 (2.7, 4)				

^a^Number of students who had never been in a choking emergency=36.

^b^Number of students who been in a choking emergency=12.

### Protocol Knowledge Before and After Playing

Focusing on the main steps of the choking protocol (Textbox 1 questions from 4 to 9), we compared the answers obtained before and after playing the game.

In Table 5, the self-impression of the knowledge of the choking protocol before and after playing the game is shown. These results are presented for the complete sample and by gender, game preference, and experience of choking scenarios. The findings in all the analyses showed an improvement after playing the game, with small effect sizes, except in the groups of males and those who liked video games, which showed moderate effect size, and in those with experience in choking scenarios, which is large.

In addition, in Table 6 and Table 7, the results of *how the students have to act in case of choking to remove the object in the throat* and *when the resuscitation protocol has to be applied* are shown. In these 2 analyses, we compared the correct answers with the incorrect answers. The results are presented for the whole sample and by gender, game preference, and experience of choking scenarios. All the indicators of the knowledge about how to act in case of a choking emergency improved after playing the game. When the same questions were analyzed by gender, game preference, and experience of the choking scenarios, all the groups achieved a significant improvement, except in the action of *removing the object from the throat*. In this case, males and those who had a previous experience of choking scenarios did not show significant improvement. In the situation of *when the resuscitation protocol has to be applied in a choking emergency*, males did not show significant improvement.

**Table 5:** Self-impression of the knowledge of the choking protocol before and after playing the game (grades from 1 to 5).

Student categories, Time of answering the questionnaire		Median (Q1, Q3)		*P* value		Effect size
**Complete sample population, N=48**			<.001		0.120	
	Before playing	3 (2, 4)				
	After playing	4 (3, 5)				
**Females, n=32**			.002		0.217	
	Before playing	3 (2.75, 3.25)				
	After playing	3 (3, 4)				
**Males, n=16**			.002		0.345	
	Before playing	3 (2, 4)				
	After playing	4 (3.75, 5)				
**Students who did not like playing video games, n=19**			.03		0.136	
	Before playing	3 (2.5, 3)				
	After playing	3 (3, 3.5)				
**Students who liked playing video games, n=29**			<.001		0.407	
	Before playing	3 (2, 4)				
	After playing	4 (4, 5)				
**Students who did not have a previous experience of a choking emergency, n=36**			<.001		0.147	
	Before playing	3 (2, 3.25)				
	After playing	4 (3, 4)				
**Students who had a previous experience of a choking emergency, n=12**			.047		0.707	
	Before playing	3 (3, 4.25)				
	After playing	4.5 (3, 5)				

**Table 6:** Statistical analysis of the responses of the action in case of choking before and after playing the game.

Student category and subcategories, Responses before the game (n)			Responses after the game (n)				*P* value
			Correct	Incorrect			
**Complete sample, N=48**							<.001
		Correct	28	0			
		Incorrect	12	8			
**Gender**							
	**Females, n=32**						<.001
		Correct	19		0		
		Incorrect	6		7		
	**Males, n=16**						.44
		Correct	9		0		
		Incorrect	6		1		
**Video game preference**							
	**Do not like, n=19**						.02
		Correct	11		0		
		Incorrect	4		4		
	**Like, n=29**					.02	
		Correct	17		0		
		Incorrect	8		4		
**Previous experience in a choking emergency**							
	**No, n=36**					.003	
		Correct	21		0		
		Incorrect	9		6		
	**Yes, n=12**					.15	
		Correct	7		0		
		Incorrect	3		2		

**Table 7:** Statistical analysis of the responses regarding the resuscitation protocol before and after playing the game.

Student category and subcategories, Responses before the game (n)			Responses after the game (n)				*P* value	
			Correct		Incorrect			
**Complete sample, N=48**								<.001
		Correct		27		1		
		Incorrect		8		12		
**Gender**								
	**Females, n=32**							<.001
		Correct		20		1		
		Incorrect		2		9		
	**Males, n=16**							.21
		Correct		7		0		
		Incorrect		6		3		
**Video game preference**								
	**Do not like, n=19**							.009
		Correct		11		1		
		Incorrect		2		5		
	**Like, n=29**							.001
		Correct		16		0		
		Incorrect		6		7		
**Previous experience in a choking emergency**								
	**No, n=36**							.003
		Correct		21		1		
		Incorrect		7		7		
	**Yes, n=12**							.015
		Correct		6		0		
		Incorrect		1		5		

## Discussion

Serious games have become a useful training technology in the health care profession and for patients to learn about the procedures involved in health care. Serious games are applicable in different fields such as surgery, odontology, cardiology, nursing, diabetes, psychology, or first-aid [16,72-74]. First-aid, triage, and mass emergency are the most popular fields, wherein games have been developed for training residents, medical doctors, or students. However, little attention has been paid to general players with no health issues. In the context of first-aid education, the majority of the proposed games have focused on the CPR protocol [53-55]. However, no games have focused on the procedure to be applied in case of choking. To overcome this limitation, in this paper, we have proposed a game to introduce the main steps of the first-aid procedure in cases of choking for the general population. This game was tested on a sample population of 48 high school students. Although tests have been done with young people, this game in our study has been designed for the general public with no age restrictions.

In the proposed game, the player acquires the role of a helper who has to save a person in a choking emergency by detecting the choking symptoms and applying the main steps of the protocol. This serious game has been designed as a set of minigames, with each minigame focused on a single concept of the protocol. Several authors have demonstrated that the use of serious minigames requires less time to master the game, which has a positive impact on the learning process. In addition, minigames make the study of a subject from different angles more encouraging [75-77]. These advantages have been exploited in this game in our study. Our minigames have very simple mechanics and the interaction is reduced to touch or drag-and-drop actions. The simplicity of this game allows the player to achieve great mastery in a short period of time. The actions reproduce some steps of the protocol that will be transmitted by applying the learning-by-doing strategy. To relate the performed actions with the protocol step, different messages are also provided to the player in the form of instructions or feedback. In addition, there is a help icon to access an animation that presents the gameplay mechanics. Time and score restrictions are imposed to pass each minigame. These restrictions increase when the game advances with the aim of creating an addiction [76].

In the tests, we observed that the players do not access the provided help icon. The players preferred a trial-and-error strategy to discover the game mechanics than the provided animations. However, in some cases, the player reached the end of the game without performing any correct action. It seems that the players prefer the trial-and-error strategy rather than the help option. To avoid this situation, we modified the help activation in such a way that if the game detects a period of time with no correct actions, the help animation is automatically activated. The idea is to reduce this trial-and-error period in order to be more effective with the minigame. In addition, if the help option is on (playing with help mode) when a new game starts, the help animation is automatically activated with no game interruption.

In the tests, we also evaluated player impressions about the game complexity, enjoyability, etc. From the results, we observed that players enjoyed the game irrespective of their gender, their preferences for video games, or their previous experience in a choking emergency. Regarding the acquired knowledge, to evaluate it, pretests and posttests were carried out. We observed that the knowledge on choking and the first-aid procedure for choking was improved through the game. Therefore, the idea of using minigames to introduce the choking concepts becomes ideal for creating an awareness of the topic in an engaging and a quick manner [78]. From these results, we considered that by focusing on protocols composed of different steps, this same strategy can be applied by simply designing serious minigames for each step and integrating all of them in a common story or in a common scenario. In this way, the whole objective of learning a protocol can be decomposed into a set of subobjectives. Although the obtained results were satisfactory, we consider that different improvements need to be done. In our study, we focused on high school students, which is a limiting factor since all of them are in the same range of ages. To overcome this limitation, our idea is to extend the study to a more general population. In addition, we want to evaluate the minigames independently. We detected some game preferences and some difficulties in some minigames but these were obtained from the visual observation and we consider that these observations are not robust enough to be included in the paper. We aim to design a new experiment to carry out this evaluation. Moreover, we have not considered any particular player’s capabilities, needs, and interests. We also want to consider players with visual impairments [70].

In conclusion, serious games are increasingly gaining attention in health care to complement and promote the training of experts in the field. However, little attention has been paid to the use of serious games as a tool to promote health in a nonexpert population. Our study proposes a serious game with the aim of educating on the first-aid procedure for choking. Our game introduces the main steps of the procedure as a set of minigames. It has been tested in a pilot study, and very promising results have been obtained. The students enjoyed the game and, more importantly, their knowledge on the first-aid for choking was found to be greatly improved. We conclude that serious games are a good strategy to promote first-aid knowledge to nonexperts.

## Abbreviations

CPR: cardiopulmonary resuscitation
